# Butterflies (Lepidoptera) of the Can Gio Mangrove Biosphere Reserve, Vietnam

**DOI:** 10.3897/BDJ.13.e165844

**Published:** 2025-10-20

**Authors:** Hiep Ngoc Dang, Hoan Duc Huynh, Trung Van Phan, Kiet Nguyen The Bui, Thao Thi Thu Luong, Vu Dang Hoang Nguyen

**Affiliations:** 1 Can Gio Protection Forest Management Board, Ho Chi Minh City, Vietnam Can Gio Protection Forest Management Board Ho Chi Minh City Vietnam; 2 Institute of Life Science, Vietnam Academy of Science and Technology, Ho Chi Minh City, Vietnam Institute of Life Science, Vietnam Academy of Science and Technology Ho Chi Minh City Vietnam

**Keywords:** biodiversity, Nymphalidae, Lycaenidae, insect, species composition

## Abstract

**Background:**

The Can Gio Mangrove Biosphere Reserve is a unique transitional ecosystem between land and sea and represents a significant wetland area in southern Vietnam. Despite being recognised as a biodiversity hotspot, its butterfly fauna remains poorly documented.

**New information:**

In this study, the Pollard Walk method was employed across 72 sampling events in 12 fixed transects, presenting the first comprehensive checklist and diversity assessment of butterflies (Lepidoptera, Rhopalocera) from this region. A total of 46 species, belonging to five families and 37 genera, were recorded through systematic field surveys conducted across 12 sites during both dry and rainy seasons from 2024 to 2025. The family Nymphalidae exhibited the highest species richness (19 species), followed by Lycaenidae (9 species), Pieridae (7 species), Hesperiidae (7 species) and Papilionidae (4 species). Two species of the most abundant were identified: *Catopsilia pomona* and *Ixias pyrene*. Our findings reveal that the butterfly community in Can Gio is moderately diverse (H’ = 1.66 ± 0.52) and exhibits seasonal variation in species composition and abundance. The study provides baseline data for future biodiversity monitoring and highlights the importance of preserving the mosaic of habitats within this mangrove biosphere reserve.

## Introduction

Butterflies (Lepidoptera, Rhopalocera) are widely recognised as important bioindicators of ecosystem health and are amongst the most studied insect groups due to their ecological roles, aesthetic appeal and sensitivity to environmental changes (McMullen 1965, Matteo et al. 2023, Irene et al. 2024). Their diversity and distribution patterns are closely linked to vegetation structure, climate and habitat quality, making them valuable indicators in biodiversity monitoring and conservation planning (Swaay CA et al. 2008).

Vietnam is considered one of the most biologically diverse countries in Southeast Asia and harbours a rich butterfly fauna, with over 1,200 species recorded to date (Monastyrskii and Devyatkin 2016). However, many regions, particularly lowland wetlands and mangrove ecosystems, remain underexplored in terms of their diversity.

The Can Gio Mangrove Biosphere Reserve, located in southern Vietnam, was designated as the country’s first UNESCO Biosphere Reserve in 2000. Spanning more than 75,000 ha of restored and natural mangrove forests, it represents a critical transition zone between freshwater and marine ecosystems. The Reserve is well known for its ecological services and biodiversity, including birds, reptiles and aquatic invertebrates (Nguyen et al. 2013, Le 2021). However, its butterfly fauna has received little scientific attention, with no comprehensive checklist or systematic diversity assessments published prior to this study.

This study aims to address that gap by documenting the species composition and variation in butterfly diversity across different habitat types within the Can Gio Mangrove Biosphere Reserve. We also highlight species of conservation concern and discuss the implications for wetland biodiversity management. The data generated from this study contribute to national biodiversity inventories and can support long-term ecological monitoring programmes in southern Vietnam.

## Materials and methods

### Study area

Coordinates: N10°24'7.21" and N10°35'26.35" Latitude; E106°56'39.33" and E106°47'15.95" Longitude.

The study was conducted in the Can Gio Mangrove Biosphere Reserve, located approximately 40 km southeast of central Ho Chi Minh City, Vietnam. The Reserve spans over 75,000 hectares and includes a mosaic of habitats such as mangrove forests, plantations, tidal flats, salt evaporation ponds and estuarine channels. The study area is characterised by a mosaic of planted mangrove forests and naturally occurring vegetation communities. The mangrove plantations primarily consist of *Rhizophora apiculata* (double-stilt mangrove) and *Ceriops* spp., which are the most extensively cultivated taxa. Additional planted species include *Kandelia candel*, *Ceriops tagal*, *Ceriops zippeliana*, *Rhizophora mucronata*, *Bruguiera sexangula*, *Intsia bijuga*, *Thespesia populnea*, *Eucalyptus* spp., *Acacia auriculiformis* and *Casuarina equisetifolia*.

In parallel with these plantations, a variety of natural plant communities and populations are present, reflecting the ecological heterogeneity of the mangrove ecosystem. Characteristic assemblages include *Sonneratia caseolaris* and *Avicennia* spp.; *Avicennia* spp., *Phoenix paludosa* and *Intsia bijuga*; *Avicennia alba* and *Sonneratia alba*; as well as *Ceriops* spp., *Lumnitzera* spp. and *Excoecaria agallocha*. Distinct monodominant populations are also observed, such as stands of *Phoenix paludosa* and *Acrostichum aureum*. Other notable communities comprise *Acrostichum aureum*, *Phoenix paludosa*, *Cordia cochinchinensis* and *Pluchea indica*; populations of *Nypa fruticans*; and *Cyperus malaccensis*, *Derris* spp. and *Acanthus* spp.

Together, these plantations and natural vegetation types create a structurally diverse and ecologically functional landscape, supporting both biodiversity conservation and coastal ecosystem services (Nguyen et al. 2020).

**Figure 1.**
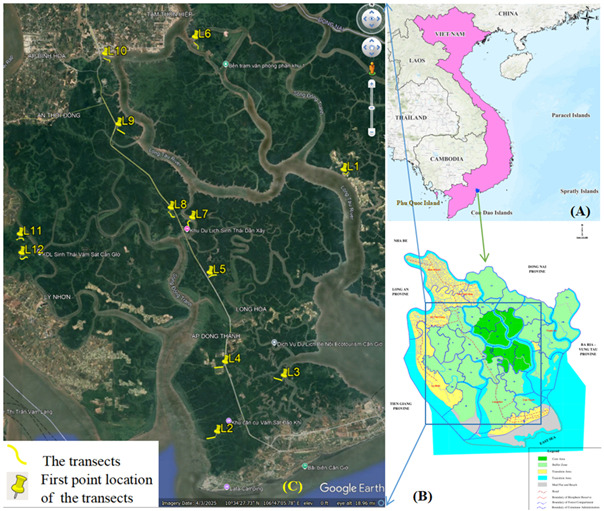
Map of the study sites. **A** Location of Can Gio Mangrove Biosphere Reserve on a map of Vietnam; **B** The study area on the Can Gio Mangrove Biosphere Reserve; **C** Ecosystems and survey areas within Can Gio Mangrove Biosphere Reserve. The map was created with QGIS software version 3.34.11. The ecosystem layer data for Google Earth Pro version 7.3.6.10201. The base map of Vietnam was sourced from GADM (Global Administrative Areas) at https://gadm.org/download_country.html

Twelve transect surveys were selected, based on habitat heterogeneity and accessibility, including areas of natural mangroves (L5, L7, L8), monoculture mangrove plantations (L1, L2, L3 and L6) and transition zones (L4, L9, L10, L11 and L12) (Fig. 1).

### Sampling effort

Six fieldwork Events were carried out between 2024 and 2025, covering both dry (November to April) and rainy (May to October) seasons at 12 transects (Fig. 1). Dates of observation on each transect are following:

- **Survey 2**:
  - 16 October 2024: L1, L6
  - 17October 2024: L7, L8, L9, L10
  - 18 October 2024: L2, L3, L4, L5
  - 19 October 2024: L11, L12.
- **Survey 3**:
  - 7 November 2024: L7, L8, L9, L10
  - 8 November 2024: L1, L6
  - 9 November 2024: L11, L12
  - 10 November 2024: L2, L3, L4, L5.
- **Survey 4**:
  - 10 January 2025: L2, L3, L4, L5
  - 11 January 2025: L7, L8, L9, L10
  - 12 January 2025: L1, L6
  - 13 January 2025: L11, L12.
- **Survey 5**:
  - 10 March 2025: L11, L12
  - 11 March 2025: L7, L8, L9, L10
  - 12 March 2025: L2, L3, L4, L5
  - 13 March 2025: L1, L6.
- **Survey 6**:
  - 23 May 2025: L2, L3, L9, L10
  - 24 May 2025: L4, L5
  - 25 May 2025: L1, L6, L7, L8
  - 26 May 2025: L11, L12.

Each study site was surveyed once per season, resulting in a total of 72 sampling events.

### Sampling description

The data on butterfly assemblages were collected using the transect method described by Pollard (1977) and modified by Spitzer et al. (1993). One full observation includes one pass. Observations occurred along each transect at a speed of approximately 20 m per min during day time from 7:30 am to 12:00 am and all observed butterflies were recorded. Species that require further identification were collected using a butterfly net.

### Species identification

Butterflies were collected and identified in the field using standard field guides (McMullen 1965, Carter 1992, Monastyrskii 2005, Bui 2007, Monastyrskii 2007, Monastyrskii 2011, Monastyrskii and Devyatkin 2016, Monastyrskii 2019, Monastyrskii 2024, Inayoshi 2025). Species-level identifications were verified by taxonomic specialists and cross-checked with the GBIF database (https://www.gbif.org/). Nomenclature follows the most recent classification by the GBIF database (GBIF - Global Biodiversity Information Facility 2025, Inayoshi 2025).

### Diversity Analysis

Butterfly diversity was compiled and annotated with habitat association from L1-L12 (Suppl. material 1), then diversity patterns were calculated for each transect using Excel, BioDiversity Professional Version 2.0 (McAleece et al. 1997), Statgraphics Version XIX Version (Nguyen 2009). The Shannon-Wiener diversity index \begin{varwidth}{50in}\begin{equation*}
            H'= -LPilnPi
        \end{equation*}\end{varwidth} where the quantity P*i* is the proportion of individuals found in the *i*^th^ species and a sample the true value of P*i* is unknown, but is estimated as \begin{varwidth}{50in}\begin{equation*}
            ni/N
        \end{equation*}\end{varwidth} (Shannon and Wiener 1963, Bibi and Ali 2013, Magurran 2013). Simpson dominance index \begin{varwidth}{50in}\begin{equation*}
            D=(n_i (n_i-1))/(N(N-1))
        \end{equation*}\end{varwidth} where *n*_*i*_ = the number of individuals in the *i*^th^ species and N = the total number of individuals (Simpson 1949, Okpiliya 2012, Magurran 2013).

## Data resources

**Figure 2.**
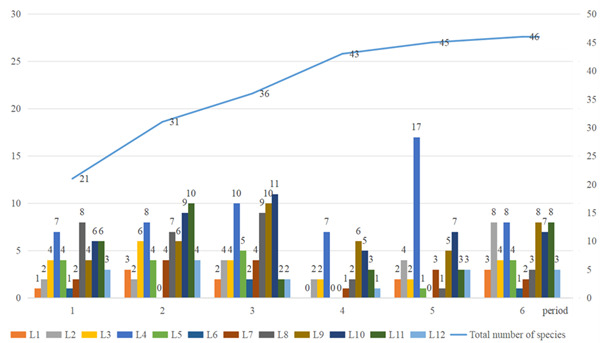
Species accumulation curve of the recorded species across six field surveys.

Our survey revealed a moderately high level of butterfly diversity in the Can Gio Mangrove Biosphere Reserve, with species composition of 46 species from five families. The number of butterfly species recorded in the study area during each survey ranged from 19 to 25 species. Through continuous survey efforts along the transects, it was observed that, from the fourth survey onwards, the number of newly-encountered species was negligible. This indicates that the survey results are approaching the estimated species richness of butterflies in the study area (Fig. 2).

**Figure 3.**
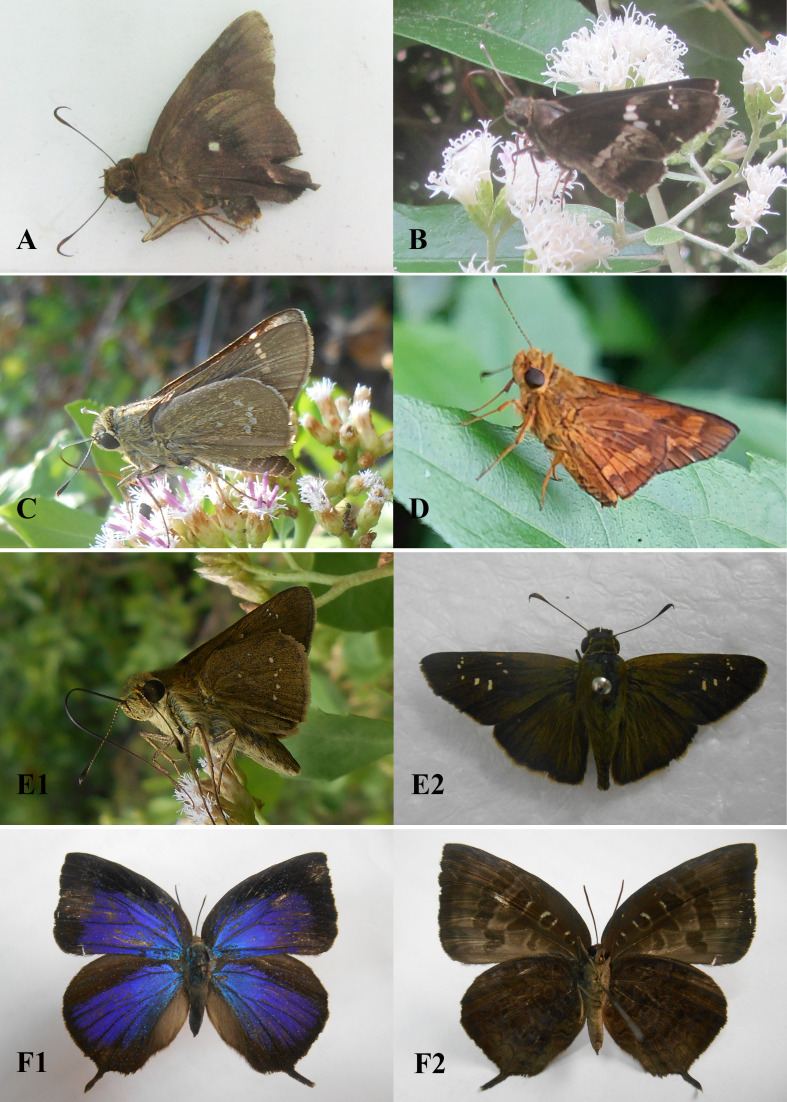
Family Hesperiidae: **A** *Hasora badra* ♂; **B** *Hyarotis adrastus*; **C** *Parnara apostata* ♀; **D** *Potanthus* sp.; **E1, E2** *Pelopidas mathias* ♂; Family Lycaenidae; **F1, F2** *Arhopala centaurus* ♀. Photos by Dang H.N.

**Figure 4.**
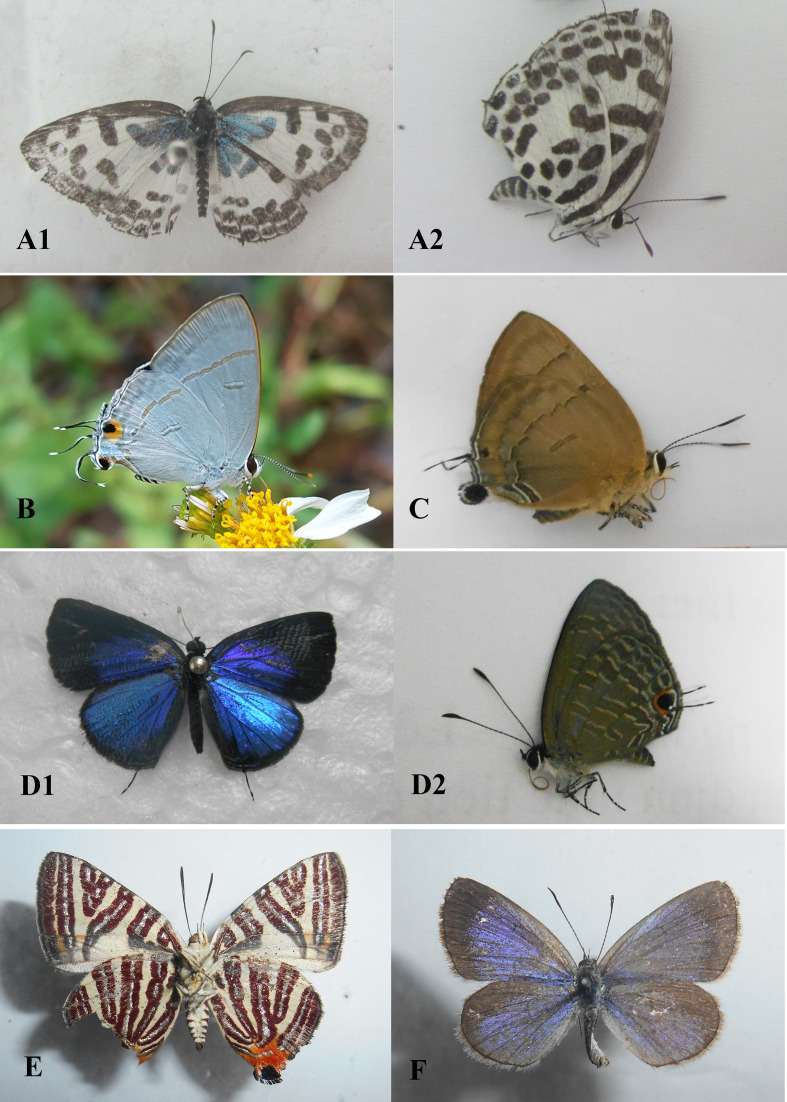
Family Lycaenidae: **A1, A2** *Castalius rosimon* ♀; **B** *Hypolycaena erylus*; **C** *Rapala pheretima* ♀; **D1, D2** *Jamides bochus* ♂; **E** *Cigaritis lohita* ♀; **F** *Zizina otis* ♂. Photos by Dang H.N.

**Figure 5.**
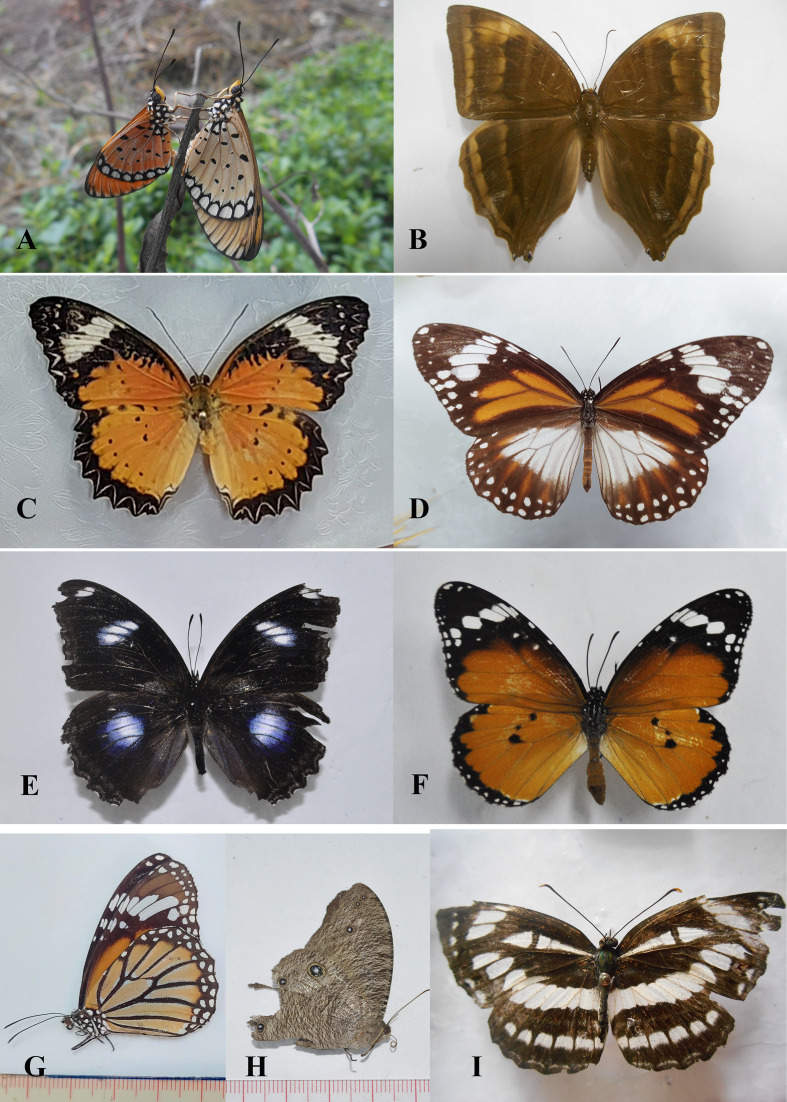
Family Nymphalidae: **A** *Acraea terpsicore* ♂(left), ♀(right); **B** *Amathusia phidippus* ♀; **C** *Cethosia cyane* ♂; D *Danaus affinis* ♀; **E** *Hypolimnas bolina* ♂; **F** *Danaus chrysippus* ♀; **G** *Danaus genutia* ♀; **H** *Melanitis leda*; **I** *Neptis hylas* ♀. Photos by Dang H.N.

**Figure 6.**
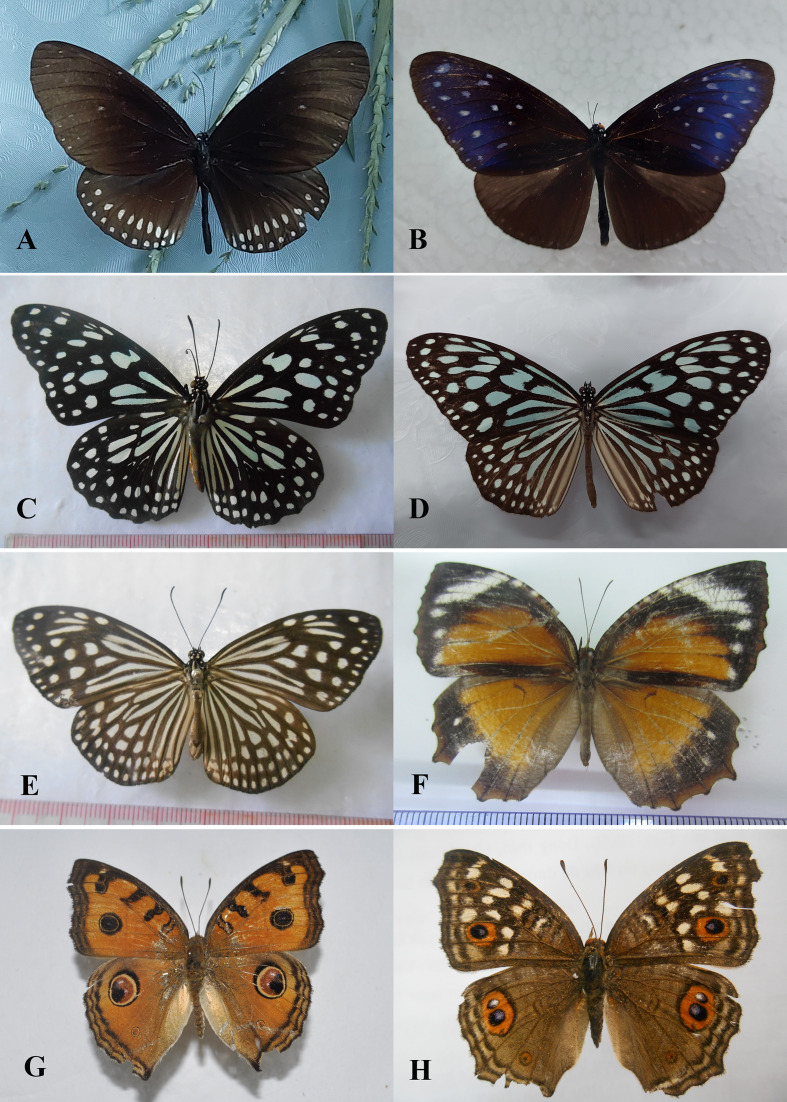
Family Nymphalidae: **A** *Euploea core* ♂; **B** *Euploea mulciber* ♂; **C** *Tirumala limniace* ♂; **D** *Ideopsis similis* ♂; **E** *Parantica agleoides* ♀; **F** *Elymnias hypermnestra* ♀; **G** *Junonia almana* ♂; **H** *Junonia lemonias* ♀. Photos by Dang H.N.

**Figure 7.**
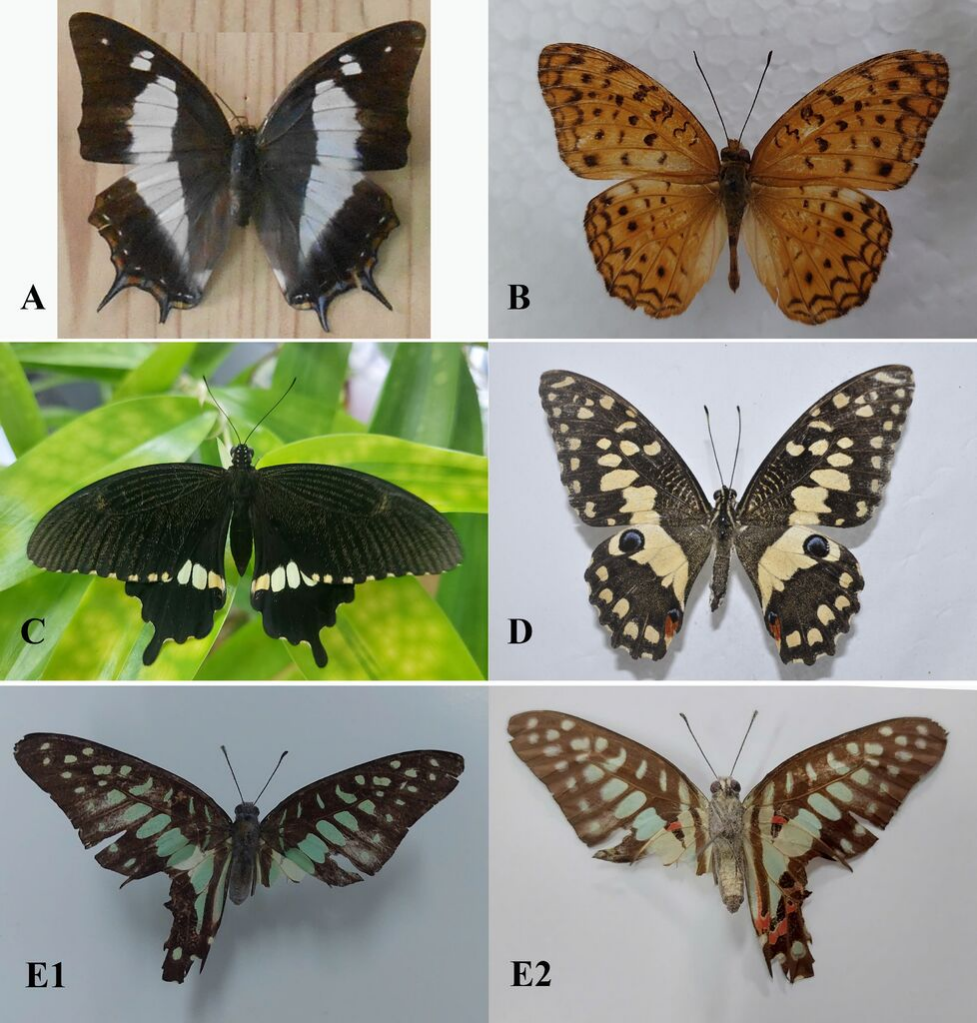
Family Nymphalidae: **A** *Polyura schreiber* ♀; **B** *Phalanta phalantha* ♂. Family Papilionidae; **C** *Papilio polytes* ♂; **D** *Papilio demoleus* ♀; **E1, E2** *Graphium doson* ♀. Photos by Dang H.N.

**Figure 8.**
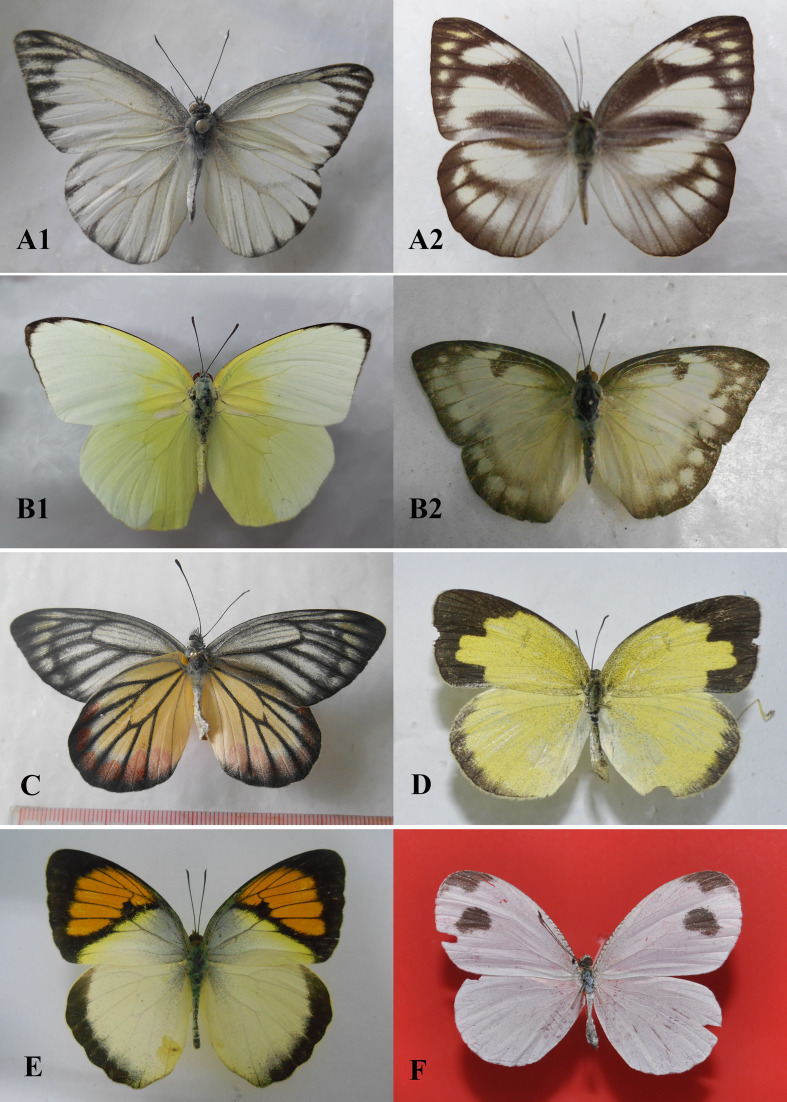
Family Pieridae: **A1, A2** *Appias olferna*; **B1, B2** *Catopsilia pomona*; **C** *Delias hyparete*; D *Eurema hecabe*; **E** *Ixias pyrene*; **F** *Leptosia nina*. Photos by Dang H.N.

Field surveys to record species composition with photos as evidence of records (Figs 3, 4, 5, 6, 7, 8) and abundance in each transect (Suppl. material 1). These data provide a foundation for calculating key biodiversity metrics, such as the Shannon-Wiener diversity index and evenness. The results aim to contribute to the ecological assessment and conservation management of the Can Gio mangrove ecosystem.

## Checklists

### Butterfly species recorded in the Can Gio Mangrove Biosphere Reserve in this study

#### Hesperiidae

Latreille, 1809

DD1CC207-F95C-56D4-8702-34C1BC634318

#### Hasora badra

(Moore, [1857])

84AA374D-7B3A-5D97-AD83-7052F6947FCA

##### Materials

- **Type status:** Other material. **Occurrence:** individualCount: 1; sex: unknown; occurrenceID: CANGIOMB0163; **Taxon:** genus: Hasora; specificEpithet: badra; scientificNameAuthorship: (Moore, [1857]); **Location:** country: Vietnam; stateProvince: Ho Chi Minh City; **Event:** eventDate: 12/01/2025

##### Notes

Recorded in Monastyrskii and Devyatkin (2016), India Biodiversity Portal (2024), GBIF - Global Biodiversity Information Facility (2025), Inayoshi (2025) and this study (Fig. 3A).

#### Hasora chromus

(Cramer, 1780)

D21F559F-D18D-5511-AC41-4D177BC2325C

##### Materials

- **Type status:** Other material. **Occurrence:** individualCount: 1; sex: unknown; occurrenceID: CANGIOMB0222; **Taxon:** genus: Hasora; specificEpithet: chromus; scientificNameAuthorship: (Cramer, 1780); **Location:** country: Vietnam; stateProvince: Ho Chi Minh City; **Event:** eventDate: 13/03/2025

##### Notes

Recorded in Bui (2007), Monastyrskii and Devyatkin (2016), India Biodiversity Portal (2024), GBIF - Global Biodiversity Information Facility (2025), Inayoshi (2025) and this study.

#### Hyarotis adrastus

(Stoll, 1780)

70EFA36A-8219-5121-9F4F-2A53F1D01A67

##### Materials

- **Type status:** Other material. **Occurrence:** individualCount: 1; sex: unknown; occurrenceID: CANGIOMB0194; **Taxon:** genus: Hyarotis ; specificEpithet: adrastus; scientificNameAuthorship: (Stoll, 1782); **Location:** country: Vietnam; stateProvince: Ho Chi Minh City; **Event:** eventDate: 11/03/2025

##### Notes

Recorded in Bui (2007), India Biodiversity Portal (2024), GBIF - Global Biodiversity Information Facility (2025), Inayoshi (2025) and this study (Fig. 3B).

#### Iambrix salsala

(Moore, 1866)

D61FEE72-590C-5CB9-9ED5-2F2BEBB33683

##### Materials

- **Type status:** Other material. **Occurrence:** individualCount: 2; sex: unknown; occurrenceID: CANGIOMB0105; **Taxon:** genus: Iambrix; specificEpithet: salsala; scientificNameAuthorship: (Moore, 1866); **Location:** country: Vietnam; stateProvince: Ho Chi Minh City; **Event:** eventDate: 09/11/2024

##### Notes

Recorded in Bui (2007), Monastyrskii and Devyatkin (2016), India Biodiversity Portal (2024), GBIF - Global Biodiversity Information Facility (2025), Inayoshi (2025) and this study.

#### Parnara apostata

(Snellen, 1880)

46A3A74F-556F-56A7-8B38-7F737D85B801

##### Materials

- **Type status:** Other material. **Occurrence:** individualCount: 2; sex: unknown; occurrenceID: CANGIOMB0018; **Taxon:** genus: Parnara; specificEpithet: apostata; scientificNameAuthorship: (Snellen, 1880); **Location:** country: Vietnam; stateProvince: Ho Chi Minh City; **Event:** eventDate: 05/09/2024

##### Notes

Recorded in Bui (2007), India Biodiversity Portal (2024), GBIF - Global Biodiversity Information Facility (2025), Inayoshi (2025) and this study (Fig. 3C).

#### Pelopidas mathias

(Fabricius, 1798)

A1B5491A-93BB-54A2-85E5-F84A3660D4A1

##### Materials

- **Type status:** Other material. **Occurrence:** individualCount: 3; sex: unknown; occurrenceID: CANGIOMB0056; **Taxon:** genus: Pelopidas; specificEpithet: mathias; scientificNameAuthorship: (Fabricius, 1798); **Location:** country: Vietnam; stateProvince: Ho Chi Minh City; **Event:** eventDate: 17/10/2024

##### Notes

Recorded in Bui (2007), Monastyrskii and Devyatkin (2016), India Biodiversity Portal (2024), GBIF - Global Biodiversity Information Facility (2025), Inayoshi (2025) and this study (Fig. 3E1, E2).

#### Potanthus sp.

1A20C8D3-CED0-58F4-8996-218761C01928

##### Materials

- **Type status:** Other material. **Occurrence:** individualCount: 1; sex: unknown; occurrenceID: CANGIOMB0075; **Taxon:** genus: Potanthus; infraspecificEpithet: sp.; **Location:** country: Vietnam; stateProvince: Ho Chi Minh City; **Event:** eventDate: 19/10/2024

##### Notes

Recorded in Monastyrskii and Devyatkin (2016), GBIF - Global Biodiversity Information Facility (2025), Inayoshi (2025) and this study (Fig. 3D).

#### Lycaenidae

Leach, 1815

F82508A0-CF8E-5883-AE22-A7E38982980B

#### Arhopala centaurus

(Fabricius, 1775)

EB8C2107-E9D5-53FC-B8B8-37BC7F2BF0D9

##### Materials

- **Type status:** Other material. **Occurrence:** individualCount: 3; sex: unknown; occurrenceID: CANGIOMB0174; **Taxon:** genus: Arhopala; specificEpithet: centaurus; scientificNameAuthorship: (Fabricius, 1775); **Location:** country: Vietnam; stateProvince: Ho Chi Minh City; **Event:** eventDate: 13/01/2025

##### Notes

Recorded in Monastyrskii and Devyatkin (2016), GBIF - Global Biodiversity Information Facility (2025), India Biodiversity Portal (2024), Inayoshi (2025) and this study (Fig. 3F1, F2).

#### Arhopala elopura

H.H. Druce, 1894

D311875B-332E-50A6-9EBE-DEFD5AC07A36

##### Materials

- **Type status:** Other material. **Occurrence:** individualCount: 2; sex: uknown; occurrenceID: CANGIOMB0106; **Taxon:** genus: Arhopala; specificEpithet: elopura; scientificNameAuthorship: Druce, 1894; **Location:** country: Vietnam; stateProvince: Ho Chi Minh City; **Event:** eventDate: 09/11/2024

##### Notes

Recorded in Bui (2007), Monastyrskii and Devyatkin (2016), India Biodiversity Portal (2024), GBIF - Global Biodiversity Information Facility (2025), Inayoshi (2025) and this study.

#### Castalius rosimon

(Fabricius, 1775)

6AFF306D-6F57-5778-AFDC-7ECB0BC0C78A

##### Materials

- **Type status:** Other material. **Occurrence:** individualCount: 2; sex: unknown; occurrenceID: CANGIOMB0118; **Taxon:** genus: Castalius; specificEpithet: rosimon; scientificNameAuthorship: (Fabricius, 1775); **Location:** country: Vietnam; stateProvince: Ho Chi Minh City; **Event:** eventDate: 10/11/2024

##### Notes

Recorded in Carter (1992), Bui (2007), Monastyrskii and Devyatkin (2016), India Biodiversity Portal (2024), GBIF - Global Biodiversity Information Facility (2025), Inayoshi (2025) and this study (Fig. 4A1, A2).

#### Cigaritis lohita

(Horsfield, 1829)

6CECC597-B1EC-5A65-9235-5E96D6987171

##### Materials

- **Type status:** Other material. **Occurrence:** individualCount: 1; sex: unknown; occurrenceID: CANGIOMB0146; **Taxon:** genus: Cigaritis ; specificEpithet: lohita; scientificNameAuthorship: (Horsfield, 1829); **Location:** country: Vietnam; stateProvince: Ho Chi Minh City; **Event:** eventDate: 11/01/2025

##### Notes

Recorded in Bui (2007), Monastyrskii and Devyatkin (2016), India Biodiversity Portal (2024), Inayoshi (2025) and this study (Fig. 4E).

#### Curetis saronis

Moore, 1877

69008441-A851-53BC-AEB7-09BA1A437E84

##### Materials

- **Type status:** Other material. **Occurrence:** individualCount: 1; sex: unknown; occurrenceID: CANGIOMB0052; **Taxon:** genus: Curetis; specificEpithet: saronis; scientificNameAuthorship: Moore, 1877; **Location:** country: Vietnam; stateProvince: Ho Chi Minh City; **Event:** eventDate: 17/10/2024

##### Notes

Recorded in Bui (2007), Monastyrskii and Devyatkin (2016), India Biodiversity Portal (2024), GBIF - Global Biodiversity Information Facility (2025), Inayoshi (2025) and this study.

#### Hypolycaena erylus

(Godart, [1823])

13035F74-C569-5B1C-8ADD-806D57EF3A73

##### Materials

- **Type status:** Other material. **Occurrence:** individualCount: 3; sex: unknown; occurrenceID: CANGIOMB0010; **Taxon:** genus: Hypolycaena ; specificEpithet: erylus; scientificNameAuthorship: (Godart, [1823]); **Location:** country: Vietnam; stateProvince: Ho Chi Minh City; **Event:** eventDate: 04/09/2024

##### Notes

Recorded in Bui (2007), Monastyrskii and Devyatkin (2016), India Biodiversity Portal (2024), GBIF - Global Biodiversity Information Facility (2025), Inayoshi (2025) and this study (Fig. 4B).

#### Jamides bochus

(Stoll 1782)

BF028B1B-B211-5739-8EF1-5B96670092B3

##### Materials

- **Type status:** Other material. **Occurrence:** individualCount: 1; sex: unknown; occurrenceID: CANGIOMB0201; **Taxon:** genus: Jamides ; specificEpithet: bochus; scientificNameAuthorship: (Stoll, 1782); **Location:** country: Vietnam; stateProvince: Ho Chi Minh City; **Event:** eventDate: 11/03/2025

##### Notes

Recorded in Monastyrskii and Devyatkin (2016), India Biodiversity Portal (2024), GBIF - Global Biodiversity Information Facility (2025), Inayoshi (2025) and this study (Fig. 4D1, D2).

#### Rapala pheretima

(Hewitson, 1863)

9571ABE6-6DF1-5C0E-BE8A-60F277D6BEF1

##### Materials

- **Type status:** Other material. **Occurrence:** individualCount: 1; sex: unknown; occurrenceID: CANGIOMB0085; **Taxon:** genus: Rapala ; specificEpithet: pheretima; scientificNameAuthorship: (Hewitson, 1863); **Location:** country: Vietnam; stateProvince: Ho Chi Minh City; **Event:** eventDate: 07/11/2024

##### Notes

Recorded in Monastyrskii and Devyatkin (2016), India Biodiversity Portal (2024), GBIF - Global Biodiversity Information Facility (2025), Inayoshi (2025) and this study (Fig. 4C).

#### Zizina otis

(Fabricius, 1787)

3D0612BB-EA39-5AD3-A7B7-C4B6078577CD

##### Materials

- **Type status:** Other material. **Occurrence:** individualCount: 50; sex: unknown; occurrenceID: CANGIOMB0007; **Taxon:** genus: Zizina ; specificEpithet: otis; scientificNameAuthorship: (Fabricius, 1787); **Location:** country: Vietnam; stateProvince: Ho Chi Minh City; **Event:** eventDate: 04/09/2024

##### Notes

Recorded in Carter (1992), Bui (2007), Monastyrskii and Devyatkin (2016), India Biodiversity Portal (2024), GBIF - Global Biodiversity Information Facility (2025), Inayoshi (2025) and this study (Fig. 4F).

#### Nymphalidae

Rafinesque, 1815

CF7EB189-04AC-5F69-B489-A955212FF569

#### Acraea terpsicore

(Linnaeus, 1758)

D9138BB9-3A6F-517E-8716-7E7E2548048C

##### Materials

- **Type status:** Other material. **Occurrence:** individualCount: 2; sex: unknown; occurrenceID: CANGIOMB0154; **Taxon:** genus: Acraea ; specificEpithet: terpsicore; scientificNameAuthorship: (Linnaeus, 1758); **Location:** country: Vietnam; stateProvince: Ho Chi Minh City; **Event:** eventDate: 11/01/2025

##### Notes

Recorded in Bui (2007), India Biodiversity Portal (2024), GBIF - Global Biodiversity Information Facility (2025), Inayoshi (2025) and this study (Fig. 5A).

#### Amathusia phidippus

(Linnaeus, 1763)

3C0F2E66-C7F5-599F-9578-456D9D1E85DE

##### Materials

- **Type status:** Other material. **Occurrence:** individualCount: 1; sex: unknown; occurrenceID: CANGIOMB0099; **Taxon:** genus: Amathusia ; specificEpithet: phidippus; scientificNameAuthorship: (Linnaeus, 1763); **Location:** country: Vietnam; stateProvince: Ho Chi Minh City; **Event:** eventDate: 09/11/2024

##### Notes

Recorded in Bui (2007), Monastyrskii and Devyatkin (2016), India Biodiversity Portal (2024), GBIF - Global Biodiversity Information Facility (2025), Inayoshi (2025) and this study (Fig. 5B).

#### Cethosia cyane

Fruhstorfer, 1912

252B5547-803E-52C2-8289-1FE6C9232BAE

##### Materials

- **Type status:** Other material. **Occurrence:** individualCount: 4; sex: male; occurrenceID: CANGIOMB0094; **Taxon:** genus: Cethosia ; specificEpithet: cyane; scientificNameAuthorship: Fruhstorfer, 1912; **Location:** country: Vietnam; stateProvince: Ho Chi Minh City; **Event:** eventDate: 08/11/2024
- **Type status:** Other material. **Occurrence:** individualCount: 2; sex: female; occurrenceID: CANGIOMB0151; **Taxon:** genus: Cethosia ; specificEpithet: cyane; scientificNameAuthorship: Drury, 1773; **Location:** country: Vietnam; stateProvince: Ho Chi Minh City; **Event:** eventDate: 11/01/2025

##### Notes

Recorded in Bui (2007), India Biodiversity Portal (2024), Inayoshi (2025) and this study (Fig. 5C).

#### Danaus affinis

(Fabricius, 1775)

0AF75B10-F825-569C-B82C-393BCA7F7098

##### Materials

- **Type status:** Other material. **Occurrence:** individualCount: 5; sex: male; occurrenceID: CANGIOMB0008; **Taxon:** genus: Danaus ; specificEpithet: affinis; scientificNameAuthorship: (Fabricius, 1775); **Location:** country: Vietnam; stateProvince: Ho Chi Minh City; **Event:** eventDate: 04/09/2024
- **Type status:** Other material. **Occurrence:** individualCount: 1; sex: female; occurrenceID: CANGIOMB0012; **Taxon:** genus: Danaus ; specificEpithet: melanippus; scientificNameAuthorship: Cramer, 1777; **Location:** country: Vietnam; stateProvince: Ho Chi Minh City; **Event:** eventDate: 25/05/2025

##### Notes

Recorded in Bui (2007), Monastyrskii and Devyatkin (2016), India Biodiversity Portal (2024), GBIF - Global Biodiversity Information Facility (2025), Inayoshi (2025) and this study (Fig. 5D).

#### Danaus chrysippus

(Linnaeus, 1758)

BB443BD7-524A-55C5-955E-63A5D09D1A6D

##### Materials

- **Type status:** Other material. **Occurrence:** individualCount: 1; sex: unknown; occurrenceID: CANGIOMB0233; **Taxon:** genus: Danaus ; specificEpithet: chrysippus; scientificNameAuthorship: ((Linnaeus, 1758); **Location:** country: Vietnam; stateProvince: Ho Chi Minh City; **Event:** eventDate: 23/05/2025

##### Notes

Recorded in Carter (1992), Bui (2007), Monastyrskii and Devyatkin (2016), India Biodiversity Portal (2024), GBIF - Global Biodiversity Information Facility (2025), Inayoshi (2025) and this study (Fig. 5F).

#### Danaus genutia

(Cramer, 1779)

3E9B2821-5773-503A-B2D0-4E5C50E545F1

##### Materials

- **Type status:** Other material. **Occurrence:** individualCount: 1; sex: unknown; occurrenceID: CANGIOMB0034; **Taxon:** genus: Danaus; specificEpithet: genutia; scientificNameAuthorship: (Cramer, 1779); **Location:** country: Vietnam; stateProvince: Ho Chi Minh City; **Event:** eventDate: 16/10/2024

##### Notes

Recorded in Bui (2007), Monastyrskii and Devyatkin (2016), India Biodiversity Portal (2024), GBIF - Global Biodiversity Information Facility (2025), Inayoshi (2025) and this study (Fig. 5G).

#### Elymnias hypermnestra

(Linnaeus, 1763)

6213784C-A3F6-59EF-9E20-498352D096F7

##### Materials

- **Type status:** Other material. **Occurrence:** individualCount: 1; sex: male; occurrenceID: CANGIOMB0089; **Taxon:** genus: Elymnias ; specificEpithet: hypermnestra; scientificNameAuthorship: (Linnaeus, 1763); **Location:** country: Vietnam; stateProvince: Ho Chi Minh City; **Event:** eventDate: 07/11/2024
- **Type status:** Other material. **Occurrence:** individualCount: 1; sex: female; occurrenceID: CANGIOMB0197; **Taxon:** genus: Elymnias ; specificEpithet: hypermnestra; scientificNameAuthorship: Linnaeus, 1763; **Location:** country: Vietnam; stateProvince: Ho Chi Minh City; **Event:** eventDate: 11/03/2025

##### Notes

Recorded in Bui (2007), Monastyrskii and Devyatkin (2016), India Biodiversity Portal (2024), GBIF - Global Biodiversity Information Facility (2025), Inayoshi (2025) and this study (Fig. 6F).

#### Euploea core

(Cramer, 1780)

448DC899-9D6F-57BA-89E6-377AD721D43B

##### Materials

- **Type status:** Other material. **Occurrence:** individualCount: 21; sex: unknown; occurrenceID: CANGIOMB0142; **Taxon:** genus: Euploea; specificEpithet: core; scientificNameAuthorship: (Cramer, 1780); **Location:** country: Vietnam; stateProvince: Ho Chi Minh City; **Event:** eventDate: 11/01/2025

##### Notes

Recorded in Bui (2007), Monastyrskii and Devyatkin (2016), India Biodiversity Portal (2024), GBIF - Global Biodiversity Information Facility (2025), Inayoshi (2025) and this study (Fig. 6A).

#### Euploea mulciber

(Cramer, 1777)

476AD9DB-A854-5278-97C9-AEC96FE8A4F0

##### Materials

- **Type status:** Other material. **Occurrence:** individualCount: 2; sex: unknown; occurrenceID: CANGIOMB0157; **Taxon:** genus: Euploea ; specificEpithet: mulciber; scientificNameAuthorship: (Cramer, 1777); **Location:** country: Vietnam; stateProvince: Ho Chi Minh City; **Event:** eventDate: 11/01/2025

##### Notes

Recorded in Carter (1992), Bui (2007), Monastyrskii and Devyatkin (2016), India Biodiversity Portal (2024), GBIF - Global Biodiversity Information Facility (2025), Inayoshi (2025) and this study (Fig. 6B).

#### Hypolimnas bolina

(Linnaeus, 1758)

8C0B2F7A-4DC8-56AF-A99F-59B41DABF402

##### Materials

- **Type status:** Other material. **Occurrence:** individualCount: 4; sex: unknown; occurrenceID: CANGIOMB0192; **Taxon:** genus: Hypolimnas ; specificEpithet: bolina; scientificNameAuthorship: (Linnaeus, 1758); **Location:** country: Vietnam; stateProvince: Ho Chi Minh City; **Event:** eventDate: 11/03/2025

##### Notes

Recorded in Carter (1992), Monastyrskii and Devyatkin (2016), India Biodiversity Portal (2024), GBIF - Global Biodiversity Information Facility (2025), Inayoshi (2025) and this study.

#### Ideopsis similis

(Linnaeus, 1758)

980A1DA6-631A-556D-BA31-07BD450E73AF

##### Materials

- **Type status:** Other material. **Occurrence:** individualCount: 1; sex: unknown; occurrenceID: CANGIOMB0216; **Taxon:** genus: Ideopsis ; specificEpithet: similis; scientificNameAuthorship: (Linnaeus, 1758); **Location:** country: Vietnam; stateProvince: Ho Chi Minh City; **Event:** eventDate: 12/03/2025

##### Notes

Recorded in Bui (2007), Monastyrskii and Devyatkin (2016), India Biodiversity Portal (2024), GBIF - Global Biodiversity Information Facility (2025), Inayoshi (2025) and this study (Fig. 6D).

#### Junonia almana

(Linnaeus, 1758)

0B746DFA-2FA6-508F-B7D6-962642FC93D1

##### Materials

- **Type status:** Other material. **Occurrence:** individualCount: 1; sex: unknown; occurrenceID: CANGIOMB0053; **Taxon:** genus: Junonia ; specificEpithet: almana; scientificNameAuthorship: (Linnaeus, 1758); **Location:** country: Vietnam; stateProvince: Ho Chi Minh City; **Event:** eventDate: 17/10/2024

##### Notes

Recorded in Bui (2007), Monastyrskii and Devyatkin (2016), India Biodiversity Portal (2024), GBIF - Global Biodiversity Information Facility (2025), Inayoshi (2025) and this study (Fig. 6G).

#### Junonia lemonias

(Linnaeus, 1758)

269E1321-76F2-52E0-BCBD-B3C1D2418915

##### Materials

- **Type status:** Other material. **Occurrence:** individualCount: 1; sex: unknown; occurrenceID: CANGIOMB0049; **Taxon:** genus: Junonia ; specificEpithet: lemonias; scientificNameAuthorship: (Linnaeus, 1758); **Location:** country: Vietnam; stateProvince: Ho Chi Minh City; **Event:** eventDate: 17/10/2024

##### Notes

Recorded in Carter (1992), Bui (2007), Monastyrskii and Devyatkin (2016), India Biodiversity Portal (2024), GBIF - Global Biodiversity Information Facility (2025), Inayoshi (2025) and this study (Fig. 6H).

#### Melanitis leda

(Linnaeus, 1758)

F961D00F-6C90-5B1E-9703-F253545D4E77

##### Materials

- **Type status:** Other material. **Occurrence:** individualCount: 1; sex: unknown; occurrenceID: CANGIOMB0128; **Taxon:** genus: Melanitis ; specificEpithet: leda; scientificNameAuthorship: (Linnaeus, 1758); **Location:** country: Vietnam; stateProvince: Ho Chi Minh City; **Event:** eventDate: 10/01/2025

##### Notes

Recorded in Bui (2007), Monastyrskii and Devyatkin (2016), India Biodiversity Portal (2024), GBIF - Global Biodiversity Information Facility (2025), Inayoshi (2025) and this study (Fig. 5H).

#### Neptis hylas

(Linnaeus, 1758)

566E78CE-BA50-5E68-B6E0-DD305B79F716

##### Materials

- **Type status:** Other material. **Occurrence:** individualCount: 1; sex: unknown; occurrenceID: CANGIOMB0086; **Taxon:** genus: Neptis ; specificEpithet: hylas; scientificNameAuthorship: (Linnaeus, 1758); **Location:** country: Vietnam; stateProvince: Ho Chi Minh City; **Event:** eventDate: 17/10/2024

##### Notes

Recorded in Bui (2007), India Biodiversity Portal (2024), GBIF - Global Biodiversity Information Facility (2025), Inayoshi (2025) and this study (Fig. 5I).

#### Phalanta phalantha

(Drury, 1773)

BB15BA94-12D9-5DD4-B654-D8C704E34BBD

##### Materials

- **Type status:** Other material. **Occurrence:** individualCount: 14; sex: unknown; occurrenceID: CANGIOMB0043; **Taxon:** genus: Phalanta ; specificEpithet: phalantha; scientificNameAuthorship: (Drury, 1773); **Location:** country: Vietnam; stateProvince: Ho Chi Minh City; **Event:** eventDate: 17/10/2024

##### Notes

Recorded in Bui (2007), Monastyrskii and Devyatkin (2016), India Biodiversity Portal (2024), GBIF - Global Biodiversity Information Facility (2025), Inayoshi (2025) and this study (Fig. 7B).

#### Polyura schreiber

(Godart, [1824])

651AFD8D-F340-5FEC-93BD-159030F8E2CA

##### Materials

- **Type status:** Other material. **Occurrence:** individualCount: 1; sex: unknown; occurrenceID: CANGIOMB0014; **Taxon:** genus: Polyura ; specificEpithet: schreiber; scientificNameAuthorship: (Godart, [1824]); **Location:** country: Vietnam; stateProvince: Ho Chi Minh City; **Event:** eventDate: 04/09/2024

##### Notes

Recorded in GBIF - Global Biodiversity Information Facility (2025), Inayoshi (2025) and this study (Fig. 7A).

#### Tirumala limniace

(Cramer, 1775)

5196C0A3-F1E7-5946-A7F2-ADD3339515F3

##### Materials

- **Type status:** Other material. **Occurrence:** individualCount: 1; sex: unknown; occurrenceID: CANGIOMB0068; **Taxon:** genus: Tirumala ; specificEpithet: limniace; scientificNameAuthorship: (Cramer, 1775); **Location:** country: Vietnam; stateProvince: Ho Chi Minh City; **Event:** eventDate: 18/10/2024

##### Notes

Recorded in Bui (2007), Monastyrskii and Devyatkin (2016), India Biodiversity Portal (2024), GBIF - Global Biodiversity Information Facility (2025), Inayoshi (2025) and this study (Fig. 6C).

#### Parantica agleoides

(Felder & Felder, 1860)

F848C0F9-69EC-5135-86D6-42F2B0C65ABB

##### Materials

- **Type status:** Other material. **Occurrence:** individualCount: 3; sex: unknown; occurrenceID: CANGIOMB0269; **Taxon:** genus: Parantica ; specificEpithet: agleoides; scientificNameAuthorship: (Felder & Felder, 1860); **Location:** country: Vietnam; stateProvince: Ho Chi Minh City; **Event:** eventDate: 11/01/2025

##### Notes

Recorded in Monastyrskii and Devyatkin (2016), GBIF - Global Biodiversity Information Facility (2025), Inayoshi (2025) and this study (Fig. 6E).

#### Papilionidae

Latreille, 1802

04C4E15D-41D7-5093-8F1B-455AFA3855EC

#### Graphium arycles

(Boisduval, 1836)

43216F06-4738-5192-9309-FB717E32F8F9

##### Materials

- **Type status:** Other material. **Occurrence:** individualCount: 1; sex: unknown; occurrenceID: CANGIOMB0005; **Taxon:** genus: Graphium; specificEpithet: arycles; scientificNameAuthorship: (Boisduval, 1836); **Location:** country: Vietnam; stateProvince: Ho Chi Minh City; **Event:** eventDate: 03/09/2024

##### Notes

Recorded in Bui (2007), Monastyrskii and Devyatkin (2016), GBIF - Global Biodiversity Information Facility (2025), Inayoshi (2025) and this study.

#### Graphium doson

(C. Felder & R. Felder, 1864)

40B71073-FFFE-509B-A164-E8318EFF9E5F

##### Materials

- **Type status:** Other material. **Occurrence:** individualCount: 1; sex: female; occurrenceID: CANGIOMB0238; **Taxon:** genus: Graphium ; specificEpithet: doson; scientificNameAuthorship: (C. Felder & R. Felder, 1864); **Location:** country: Vietnam; stateProvince: Ho Chi Minh City; **Event:** eventDate: 23/05/2025

##### Notes

Recorded in Bui (2007), Monastyrskii and Devyatkin (2016), India Biodiversity Portal (2024), GBIF - Global Biodiversity Information Facility (2025), Inayoshi (2025) and this study (Fig. 7E1, E2).

#### Papilio demoleus

Linnaeus, 1758

43AF41AA-A2B2-55A6-9F0C-49D8609F775D

##### Materials

- **Type status:** Other material. **Occurrence:** individualCount: 1; sex: female; occurrenceID: CANGIOMB0191; **Taxon:** genus: Papilio ; specificEpithet: demoleus; scientificNameAuthorship: Linnaeus, 1758; **Location:** country: Vietnam; stateProvince: Ho Chi Minh City; **Event:** eventDate: 11/03/2025

##### Notes

Recorded in Bui (2007), Monastyrskii and Devyatkin (2016), India Biodiversity Portal (2024), GBIF - Global Biodiversity Information Facility (2025), Inayoshi (2025) and this study (Fig. 7D).

#### Papilio polytes

Linnaeus, 1758

77339005-C209-51E0-BB1D-2562DA4BC862

##### Materials

- **Type status:** Other material. **Occurrence:** individualCount: 1; sex: male; occurrenceID: CANGIOMB0059; **Taxon:** genus: Papilio ; specificEpithet: polytes; scientificNameAuthorship: Linnaeus, 1758; **Location:** country: Vietnam; stateProvince: Ho Chi Minh City; **Event:** eventDate: 18/10/2024
- **Type status:** Other material. **Occurrence:** individualCount: 1; sex: female; occurrenceID: CANGIOMB0259; **Taxon:** genus: Papilio ; specificEpithet: polytes; scientificNameAuthorship: Linnaeus, 1758; **Location:** country: Vietnam; stateProvince: Ho Chi Minh City; **Event:** eventDate: 25/05/2025

##### Notes

Recorded in Carter (1992), Bui (2007), Monastyrskii and Devyatkin (2016), India Biodiversity Portal (2024), GBIF - Global Biodiversity Information Facility (2025), Inayoshi (2025) and this study (Fig. 7C).

#### Pieridae

Swainson, 1820

2090EDE9-F11D-5742-8D9C-FD3DD39DDE18

#### Appias olferna

Swinhoe, 1890

E36313FA-0EA2-5E66-AC93-D94BDE36FDA0

##### Materials

- **Type status:** Other material. **Occurrence:** individualCount: 15; sex: female; occurrenceID: CANGIOMB0019; **Taxon:** genus: Appias ; specificEpithet: olferna; scientificNameAuthorship: Swinhoe, 1890; **Location:** country: Vietnam; stateProvince: Ho Chi Minh City; **Event:** eventDate: 03/9/2024
- **Type status:** Other material. **Occurrence:** individualCount: 26; sex: male; occurrenceID: CANGIOMB0247; **Taxon:** genus: Appias; specificEpithet: libythea; scientificNameAuthorship: (Fabricius, 1775); **Location:** country: Vietnam; stateProvince: Ho Chi Minh City; **Event:** eventDate: 24/05/2025

##### Notes

Recorded in Bui (2007), India Biodiversity Portal (2024), GBIF - Global Biodiversity Information Facility (2025), Inayoshi (2025) and this study (Fig. 8A1, A2).

#### Catopsilia pomona

(Fabricius, 1775)

FEB7FBF5-AB96-5386-AD2C-3B9592ECBDF9

##### Materials

- **Type status:** Other material. **Occurrence:** individualCount: 143; sex: male; occurrenceID: CANGIOMB0176; **Taxon:** genus: Catopsilia; specificEpithet: pomona; scientificNameAuthorship: (Fabricius, 1775); **Location:** country: Vietnam; stateProvince: Ho Chi Minh City; **Event:** eventDate: 19/10/2025
- **Type status:** Other material. **Occurrence:** individualCount: 52; sex: female; occurrenceID: CANGIOMB0177; **Taxon:** genus: Catopsilia; specificEpithet: pomona; scientificNameAuthorship: (Fabricius, 1775); **Location:** country: Vietnam; stateProvince: Ho Chi Minh City; **Event:** eventDate: 10/03/2025

##### Notes

Recorded in Bui (2007), Monastyrskii and Devyatkin (2016), India Biodiversity Portal (2024), GBIF - Global Biodiversity Information Facility (2025), Inayoshi (2025) and this study (Fig. 8B1, B2).

#### Catopsilia pyranthe

(Linnaeus, 1758)

CE7D1D90-291B-55C9-8E23-6B2CF4EAB231

##### Materials

- **Type status:** Other material. **Occurrence:** individualCount: 1; sex: unknown; occurrenceID: CANGIOMB0111; **Taxon:** genus: Catopsilia; specificEpithet: pyranthe; scientificNameAuthorship: (Linnaeus, 1758); **Location:** country: Vietnam; stateProvince: Ho Chi Minh City; **Event:** eventDate: 10/11/2024

##### Notes

Recorded in Bui (2007), Monastyrskii and Devyatkin (2016), India Biodiversity Portal (2024), GBIF - Global Biodiversity Information Facility (2025), Inayoshi (2025) and this study.

#### Delias hyparete

(Linnaeus, 1758)

0DB47AF5-2979-5925-B024-D9AE3BEB65DD

##### Materials

- **Type status:** Other material. **Occurrence:** individualCount: 2; sex: unknown; occurrenceID: CANGIOMB0087; **Taxon:** genus: Delias ; specificEpithet: hyparete; scientificNameAuthorship: (Linnaeus, 1758); **Location:** country: Vietnam; stateProvince: Ho Chi Minh City; **Event:** eventDate: 17/10/2024

##### Notes

Recorded in Bui (2007), Monastyrskii and Devyatkin (2016), India Biodiversity Portal (2024), GBIF - Global Biodiversity Information Facility (2025), Inayoshi (2025) and this study (Fig. 8C).

#### Eurema hecabe

(Linnaeus, 1758)

F19AA1C3-0DBF-52EC-BFAC-60861F0EB58B

##### Materials

- **Type status:** Other material. **Occurrence:** individualCount: 9; sex: unknown; occurrenceID: CANGIOMB0230; **Taxon:** genus: Eurema ; specificEpithet: hecabe; scientificNameAuthorship: (Linnaeus, 1758); **Location:** country: Vietnam; stateProvince: Ho Chi Minh City; **Event:** eventDate: 23/05/2025

##### Notes

Recorded in Bui (2007), Monastyrskii and Devyatkin (2016), India Biodiversity Portal (2024), GBIF - Global Biodiversity Information Facility (2025), Inayoshi (2025) and this study (Fig. 8D).

#### Ixias pyrene

(Linnaeus, 1764)

4F6F604F-78C9-5C22-8F05-92EB1B7EA48F

##### Materials

- **Type status:** Other material. **Occurrence:** individualCount: 40; sex: unknown; occurrenceID: CANGIOMB0195; **Taxon:** genus: Ixias ; specificEpithet: pyrene; scientificNameAuthorship: (Linnaeus, 1764); **Location:** country: Vietnam; stateProvince: Ho Chi Minh City; **Event:** eventDate: 07/11/2025

##### Notes

Recorded in Monastyrskii and Devyatkin (2016), India Biodiversity Portal (2024), GBIF - Global Biodiversity Information Facility (2025), Inayoshi (2025) and this study (Fig. 8E).

#### Leptosia nina

(Fabricius, 1793)

F9186DE6-57E6-59DA-8799-6A06B351F4E6

##### Materials

- **Type status:** Other material. **Occurrence:** individualCount: 14; sex: unknown; occurrenceID: CANGIOMB0170; **Taxon:** genus: Leptosia ; specificEpithet: nina; scientificNameAuthorship: (Fabricius, 1793); **Location:** country: Vietnam; stateProvince: Ho Chi Minh City; **Event:** eventDate: 13/01/2025

##### Notes

Recorded in Bui (2007), Monastyrskii and Devyatkin (2016), India Biodiversity Portal (2024), GBIF - Global Biodiversity Information Facility (2025), Inayoshi (2025) and this study (Fig. 8F).

## Analysis

The average Simpson index (D) in the total survey was 0.29 ± 0.05 close to 0, demonstrating that there was dominance of some species amongst the butterflies in the surveyed transects (Table 1). Of which, L2 had the highest dominance, followed by L1, L7 and L12 with D index values of 0.74, 0.44, 0.36 and 0.35, respectively. This shows that the butterfly community was dominated by some species including *C. pomona*, *I. pyrene*, *A. libythea* etc., in which, *C. pomona* appeared in and was dominant in most of the surveyed transects with 711 encountered individuals.

Butterfly diversity varied amongst transects, with the highest (1.93–2.53) at the transition zones (transect L4, L9, L10 and L11) (Table 1). In contrast, sites dominated by monoculture mangrove plantations (L1, L2 and L6) had lowest diversity (0.07–1.13). The areas of natural mangroves (L7, L8) were average in diversity (1.68–1.73). Overall, butterfly diversity was moderately high, with a mean Shannon-Wiener index (H’) of 1.66 ± 0.15.

The dominance of the family Nymphalidae is consistent with patterns observed in other Southeast Asian tropical lowland forests (Monastyrskii 2011). Their ecological versatility, broader host plant range and adaptability to a variety of microhabitats likely contribute to their high representation. Conversely, the relatively lower species diversity in monoculture mangrove plantations may reflect habitat simplification and the absence of larval host plants or nectar sources.

## Discussion

Although the present study provides the first comprehensive checklist for Can Gio Mangrove Biosphere Reserve, we acknowledge that butterfly diversity may still be under-represented due to methodological limitations (e.g. reliance on visual surveys and daytime sampling). Future studies incorporating traps, longer survey durations and larval host plant assessments would help provide a more complete picture.

This study presents the first comprehensive assessment of butterfly diversity in the Can Gio Mangrove Biosphere Reserve, highlighting a moderately high level of species abundance across five families. The baseline data generated herein can support future biodiversity monitoring, ecological restoration and conservation planning in mangrove and lowland wetland systems in Vietnam. We recommend ongoing monitoring, integration of additional sampling methods and targeted habitat management to maintain and enhance butterfly diversity in the Reserve.

In conclusion, the Can Gio Mangrove Biosphere Reserve supports a diverse assemblage of butterflies. Protecting the mosaic of habitats — particularly transitional and edge areas— is essential for sustaining Rhopalocera diversity in this important wetland landscape.

## Supplementary Material

XML Treatment for Hesperiidae

XML Treatment for Hasora badra

XML Treatment for Hasora chromus

XML Treatment for Hyarotis adrastus

XML Treatment for Iambrix salsala

XML Treatment for Parnara apostata

XML Treatment for Pelopidas mathias

XML Treatment for Potanthus sp.

XML Treatment for Lycaenidae

XML Treatment for Arhopala centaurus

XML Treatment for Arhopala elopura

XML Treatment for Castalius rosimon

XML Treatment for Cigaritis lohita

XML Treatment for Curetis saronis

XML Treatment for Hypolycaena erylus

XML Treatment for Jamides bochus

XML Treatment for Rapala pheretima

XML Treatment for Zizina otis

XML Treatment for Nymphalidae

XML Treatment for Acraea terpsicore

XML Treatment for Amathusia phidippus

XML Treatment for Cethosia cyane

XML Treatment for Danaus affinis

XML Treatment for Danaus chrysippus

XML Treatment for Danaus genutia

XML Treatment for Elymnias hypermnestra

XML Treatment for Euploea core

XML Treatment for Euploea mulciber

XML Treatment for Hypolimnas bolina

XML Treatment for Ideopsis similis

XML Treatment for Junonia almana

XML Treatment for Junonia lemonias

XML Treatment for Melanitis leda

XML Treatment for Neptis hylas

XML Treatment for Phalanta phalantha

XML Treatment for Polyura schreiber

XML Treatment for Tirumala limniace

XML Treatment for Parantica agleoides

XML Treatment for Papilionidae

XML Treatment for Graphium arycles

XML Treatment for Graphium doson

XML Treatment for Papilio demoleus

XML Treatment for Papilio polytes

XML Treatment for Pieridae

XML Treatment for Appias olferna

XML Treatment for Catopsilia pomona

XML Treatment for Catopsilia pyranthe

XML Treatment for Delias hyparete

XML Treatment for Eurema hecabe

XML Treatment for Ixias pyrene

XML Treatment for Leptosia nina

2180BB8B-0D46-54F4-9727-8D759A6AC958 10.3897/BDJ.13.e165844.suppl1 Supplementary material 1 Abundance of butterfly species across transect sites Data type occurrences Brief description Butterfly abundance data for the transects L1-L12. File: oo_1423050.xlsx https://binary.pensoft.net/file/1423050 Dang Ngoc Hiep, Huynh Duc Hoan

## Figures and Tables

**Table 1.** Biological index by survey transects from L1 to L12. Note: areas of natural mangroves (L5, L7, L8), monoculture mangrove plantations (L1, L2, L3 and L6) and transition zone (L4, L9, L10, L11 and L12)

**Index**	**L1**	**L2**	**L3**	**L4**	**L5**	**L6**	**L7**	**L8**	**L9**	**L10**	**L11**	**L12**	**AVG**	**SE**	**SD**
H’	1.13	0.70	1.79	2.53	2.06	1.04	1.68	1.73	1.93	1.98	2.03	1.35	1.66	0.15	0.52
D	0.44	0.74	0.22	0.11	0.12	0.17	0.36	0.30	0.22	0.25	0.18	0.35	0.29	0.05	0.17
H’dry season	1.16	0.30	1.61	2.08	2.01	0.69	1.50	1.61	1.79	1.99	2.09	1.61	1.54	0.16	0.56
H’rainy season	0.82	1.81	1.65	2.37	1.03	0.69	1.30	1.73	1.79	1.83	1.64	0.77	1.45	0.15	0.52
